# Training needs in data, informatics and technical skills in the US governmental public health workforce

**DOI:** 10.3389/fpubh.2026.1766682

**Published:** 2026-01-30

**Authors:** Sripriya Rajamani, Divya Rupini Gunashekar, Umesh Ghimire, Jonathon P. Leider, Brian E. Dixon

**Affiliations:** 1 School of Nursing, University of Minnesota, Minneapolis, MN, United States; 2 Institute for Health Informatics, University of Minnesota, Minneapolis, MN, United States; 3 School of Public Health, University of Minnesota, Minneapolis, MN, United States; 4 Department of Health Policy & Management, Fairbanks School of Public Health, Indiana University Indianapolis, Indianapolis, IN, United States; 5 Center for Biomedical Informatics, Regenstrief Institute; Indianapolis, IN, United States; 6 Center for Health Information and Communications, Richard L. Roudebush VA Medical Center, Health Systems Research Division, Department of Veterans Affairs, Indianapolis, IN, United States

**Keywords:** digital public health, informatics workforce, public health informatics, public health workforce development, technical skills assessment

## Abstract

**Introduction:**

A workforce with data and technology skills is vital to support digital transformation in public health (PH). Using US-based 2024 Public Health Workforce Interests and Needs Survey (PH WINS) national survey data, this study examined informatics/data skills gaps and training needs of governmental PH workers.

**Methods:**

2024 PH WINS included data from 48 state health agencies-central office (SHA-CO), 34 large local health departments (LHD) from Big Cities Health Coalition (BCHC), and 1,144 other LHDs. Analysis focused on non-supervisory Tier 1 respondents, their education and position. PH workforce classifications (*N* = 77) were grouped into five categories. Training needs defined as discordance between job importance and skill need (high importance/low skill). Gaps were assessed for six data/informatics-related questions using SAS v9.4, along with time and resource needs.

**Results:**

Of 56,595 respondents, 72% (*N* = 40,914) worked in Tier 1 roles. Across three PH settings, half (45–54%) had BS/MS/PhD degrees, but not in public health. Half (50–52%) worked for 0–5 years in current position; one-third had 0–5 years in PH practice. Data/informatics-related roles reported least technical skill gaps across all settings: SHA-CO (8.1%), BCHC (6.9%) and LHD (8.1%). For addressing training needs, nurses self-reported most need for time, and data/informatics roles indicated high resource needs (22% BCHC; 20% LHDs). Analysis showed significant skills gaps (*p* < 0.05) in SHA-CO for all the six data/informatics topics.

**Discussion:**

Results highlight a widespread training need in incumbent PH workforce to address the data-related/technical skills gaps. Multi-pronged training approaches are critical to building a data and informatics-savvy PH workforce.

## 1 Introduction

Data is the backbone of public health and is increasingly important given the growing adoption of digital tools to support various public health services and programs. The 2025 core data functions and capabilities report from the Council of State and Territorial Epidemiologists (CSTE) (1) advocates the need for a skilled workforce capable of systems modernization aligning with evolving data standards and the need for training to build data analytic capabilities. The CSTE report in 2024 had also pointed out to informatics as a growth area with data modernization (2). The 2024 informatics profile from the National Association of County and City Health Officials (NACCHO) identified informatics as an area of staff training needs (3). This assessment highlighted the fact that more than half (61%) of local health departments (LHD) had no staff dedicated to informatics services. The limited informatics staff capacity in small and rural local health departments is attributed to a growing digital divide and a limiting factor in implementing data modernization projects (4).

The Public Health Data Strategy (PHDS) (5) was launched by the US Centers for Disease Control and Prevention (CDC) to modernize the nation’s public health data systems. This aims to provide timely and actionable information by addressing gaps in data and reducing the complexity of data exchange. The PHDS recognizes workforce as an important component and supports the Workforce Acceleration Initiative (WAI) (6). This initiative accelerates and bolsters various information system improvements by providing the needed data and technology experts for placement in public health agencies. As these placements are fulfilled by experts who are novice to public health, the WAI also provides core training in public health and public health informatics.

Building the Public Health Informatics (PHI) workforce was among the key strategies to strengthen the U.S. public health information infrastructure, per recommendations from the American College of Medical Informatics (ACMI) (7). Public health education in majority of academic programs does not include informatics and data science as one of the core competencies (8). Additionally, there is paucity of interprofessional training between public health and information technology (IT) (8). This scenario has led to many public health agencies lacking a skilled workforce needed for systems modernization projects. The NACCHO report underscored the need to upskill the incumbent governmental public health workforce with informatics and data science training (3).

Prior research highlighted the scarcity of PHI specialists who accounted for less than 2% of the governmental public health workforce (9). A gap analysis of skills related to informatics-related tasks was done and the study noted that the PHI specialists reported lower skill gaps in data/informatics areas when compared to other public health roles. Given the growing importance of an informatics-savvy workforce, it is vital to assess and understand the current state of data and informatics-related skills needs in the incumbent public health workforce.

The Public Health Workforce Interests and Needs Survey (PH WINS) (10), a nationally representative survey of the governmental public health workforce, can facilitate the understanding of skill needs related to public health practice in the digital age. This survey has self-reported data on skills and their importance to a specific job role across multiple job classifications and various public health settings. The objective of this research is to analyze the recent PH WINS data from 2024 to generate insights on data and informatics-related training needs across job classifications and public health settings. The overarching goal is to advocate for targeted training programs that prepare the current public health students with adequate digital skills and upskill the current workforce for adaptation to the digital age, recognizing the gaps identified by various roles.

## 2 Methods

The PH WINS survey aims to measure the strengths and gaps related to training, recruitment, and retention in the US governmental public health workforce. This is a collaboration of the de Beaumont Foundation and the Association of State and Territorial Health Officials. It is the only nationally representative data currently available on the governmental public health workforce and has been conducted 4 times to date (2014, 2017, 2021, 2024). The survey instrument is publicly available (11), and the data is made available to select group of researchers. Detailed methodology has been published by the PH WINS research team (12), and in the 2024 survey cycle, a nationally representative sample of small LHDs was achieved for the first time, as all state and local public health departments in the US had the opportunity for participation.

2024 PH WINS data included responses from 48 state health agencies-central office (SHA-CO), 34 large LHDs who are members of the Big Cities Health Coalition (BCHC), and 1,144 other LHDs (12). The dataset has responses from 56,595 eligible respondents (37% response rate). A list of 77 public health workforce classifications was grouped into five categories: (1) Administrative; (2) Clinical/Behavioral (BH)/Social Services (SS); (3) Data/Informatics; (4) Nurses and (5) Public Health Sciences. A small subset (<1%) of classifications had overlap with supervisory roles and so excluded from further analysis. The category of Data/Informatics was composed using 9 data-related positions, including Epidemiologist; Information Systems Manager/Information Technology Specialist; Public Health Informatics Specialist; Statistician; Data or Research Analyst; Application/Software Developer; Data Scientist; Other Data/Computer Scientist; and Database Manager/Data Storage Architect. This category is unique from other PH WINS analyses. This group was created based on insights from prior research (9) and based on study team discussion that this group has some overlapping informatics functionalities in public health practice.

The survey asked respondents to self-rate their current skills and the importance of those skills for their job. Training needs were defined as discordance between job importance and skill need (high importance/low skill) identified by respondents. Skill gaps, which was considered equivalent to training needs in this study, were assessed for 6 questions related to digital / technical skills. These included: (1) Technical skills specific to my programmatic area; (2) Identify Appropriate Sources of Data and Information to Assess the Health of a Community; (3) Collect valid data for use in decision making; (4) Participate in Quality Improvement Processes for Agency Programs and Services; (5) Identify evidence-based approaches to address public health issues; and (6) Content knowledge specific to my programmatic area. These questions were chosen based on guidance from prior studies (9, 13) and based on discussions by the study team.

In addition, the time and resources provided by agencies to address training needs was assessed. “My agency provides me with time to address my training needs” was the question pertaining to time, and one on resources was “My agency provides me with resources to address my training needs.” The agree and strongly agree responses were combined into one category. Likewise, the disagree and strongly disagree were combined into one category. Additional analysis focused on the disagree/strongly disagree category as they indicate the need for time and resources for training.

The training needs related to digital /technical skills were explored in detail. National weights provided with the 2024 PH WINS survey data were used in various analyses to derive weighted percentiles in the results. These weights account for the complex survey design and the differential non-responses across employees and settings. Differences in skill gaps between job roles were analyzed using the design-adjusted Rao-Scott *χ*^2^ test.

Survey respondents selected their tier based on their position: Tier 1 (non-supervisors); Tier 2 (supervisors and managers) and Tier 3 (Executives). This study analyzed the non-supervisory tier (Tier 1) respondents representing frontline PH workers as they are the largest segment of the workforce and includes technical staff who work on modernization activities. The results were organized by job classification categories and by public health settings (SHA-CO; BCHC and LHD). All analyses were performed with SAS 9.4 (SAS Institute, Inc., Carey, NC, USA), and graphs created with Microsoft Excel.

## 3 Results

**Figure 1:**
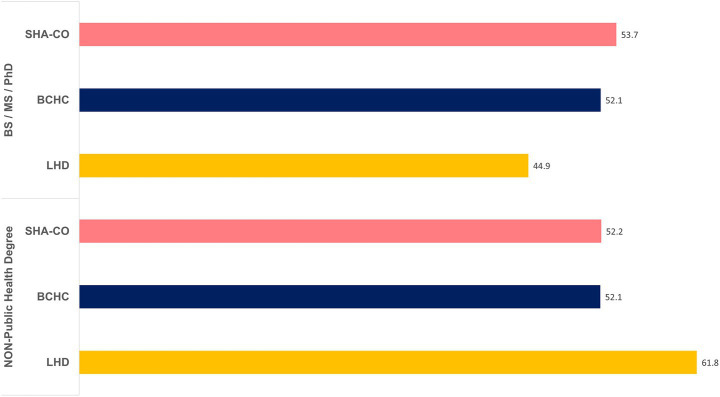
Education and tenure in public health among Tier 1 governmental public health workers.

Of the 56,595 respondents, about 72% (*N* = 40,914) indicated they worked in Tier 1 non-supervisory roles. Figure 1 portrays the education of Tier 1 governmental public health workers and additionally, if they had a public degree. Across the various PH settings, those who had Bachelors/Masters/Doctoral were approximately half of respondents (54% in SHA-CO; 52% in BCHC; 45% in LHDs). But those degrees were not in public health for 52–62% of Tier 1 workers across the three PH settings (SHA-CO, BCHC and LHD).

**Figure 2:**
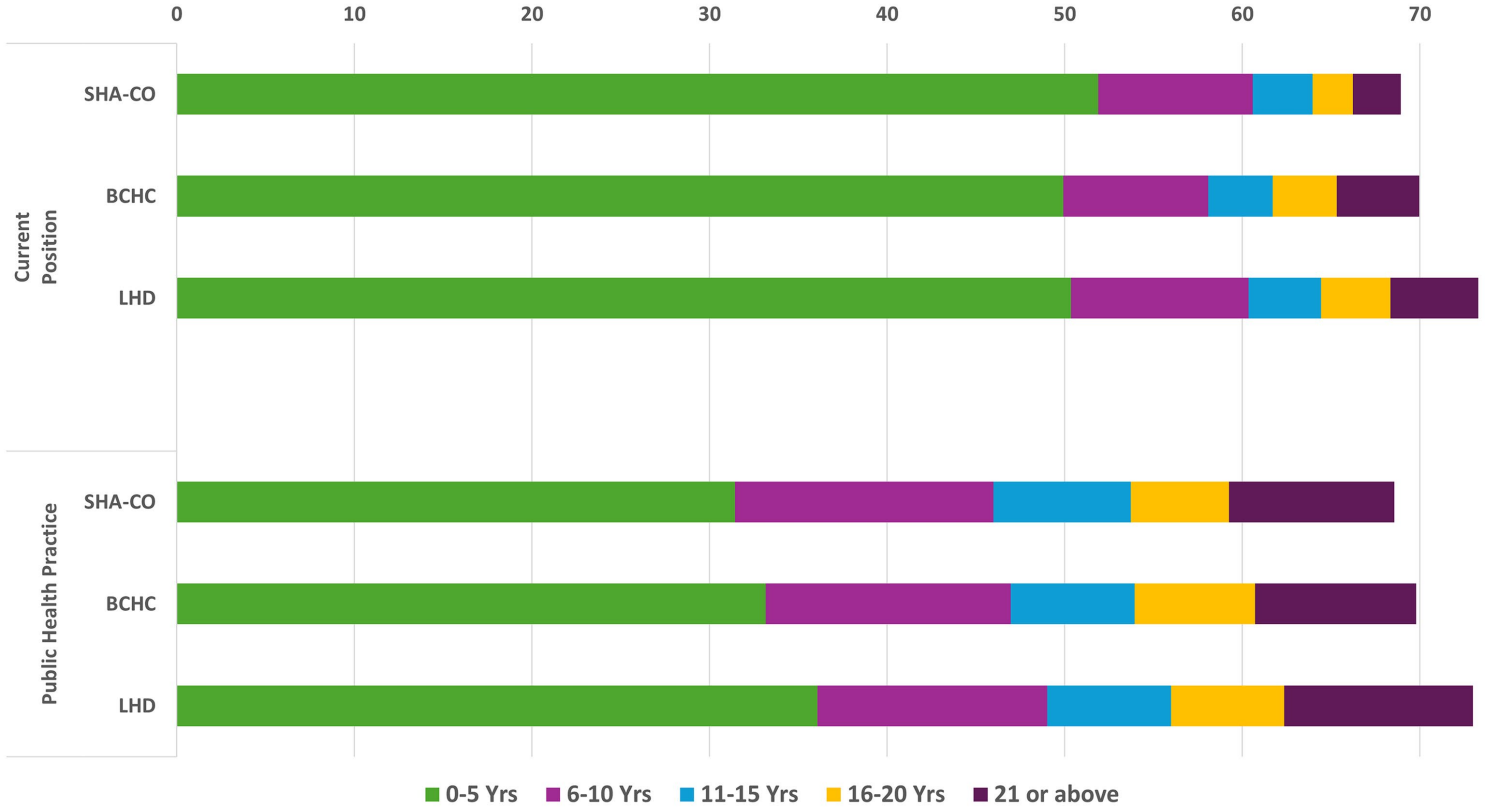
Duration in current position and in public health practice among Tier 1 governmental public health workers.

Figure 2 presents data related to years of tenure in public health. In response to the question on duration in their current position, close to half of Tier 1 workers indicated less than 5 years (52% in SHA-CO; 50% in BCHC and 50% in LHDs). For the question on length in public health practice (any agency, any position), approximately one-third of respondents had experience of less than 5 years (31% in SHA-CO; 33% in BCHC and 36% in LHDs).

Table 1 presents the training needs for all six data/informatics related topics: technical skills, appropriate sources of data, valid data for decision making, quality improvement processes, evidence-based approaches, and content knowledge skills. The Tier 1 respondents included all five job categories across Administrative; Clinical/Behavioral/Social Services; Data/Informatics; Nurses and Public Health Science. In response to the prompt, “Technical skills specific to my programmatic area,” PH workers in data/informatics-related roles reported the fewest training needs across all the three settings: SHA-CO (8.1%), BCHC (6.9%), and LHD (8.1%). In the SHA-CO setting, nurses self-reported the highest skill gap with approximately one-fifth (17.3%), indicating training needs. This was followed by administrative roles (13%) and clinical roles (12%) with skill gaps. In the BCHC setting, the job category with the highest skill gap was clinical roles (14.1%), followed by administrative roles (13.4%). Administrative roles also reported the highest skill gap (13.3%) in the LHD setting, followed by clinical roles (13%).

**Table 1:** Training needs for data and informatics-related questions by public health settings and job classifications.

Settings and roles	Technical skills specific to my programmatic area		Identify appropriate sources of data and information to assess the health of a community		Collect valid data for use in decision making		Participate in quality improvement processes for agency programs and services		Identify evidence-based approaches to address public health issues		Content knowledge specific to my programmatic area	
	*n*	W%(SE)	*n*	W%(SE)	*n*	W%(SE)	*n*	W%(SE)	*n*	W%(SE)	*n*	W%(SE)
SHA-CO												
Administrative	316	13.04 (0.80)	360	24.74 (1.34)	335	14.61 (0.85)	504	27.13 (1.19)	352	25.44 (1.37)	369	14.64 (0.81)
Clinical/BH/SS	82	12.32 (1.46)	92	16.59 (1.77)	91	13.59 (1.52)	152	28.35 (2.19)	92	16.23 (1.74)	68	10.16 (1.32)
Data/Informatics	177	8.06 (0.67)	158	8.94 (0.79)	109	5.50 (0.59)	328	23.26 (1.28)	187	12.79 (1.01)	194	9.11 (0.71)
Nurse	129	17.25 (1.62)	117	14.69 (1.46)	120	13.68 (1.36)	182	26.70 (2.00)	111	14.41 (1.48)	133	18.23 (1.66)
Public Health Science	429	9.92 (0.52)	502	15.26 (0.71)	537	13.82 (0.64)	796	24.90 (0.88)	529	15.46 (0.70)	472	11.06 (0.54)
*p*	*<0.0001*		*<0.0001*		*<0.0001*		*0.0005*		*0.0164*		*<0.0001*	
BCHC												
Administrative	159	13.37 (1.23)	184	20.12 (1.69)	148	12.22 (1.14)	260	25.51 (1.83)	187	21.09 (1.72)	173	15.88 (1.45)
Clinical/BH/SS	139	14.09 (1.53)	149	15.12 (1.57)	131	14.13 (1.63)	201	23.99 (2.06)	164	15.91 (1.61)	126	12.99 (1.53)
Data/Informatics	43	6.89 (1.24)	44	9.34 (1.81)	41	8.69 (1.73)	89	19.95 (2.52)	52	12.56 (2.27)	45	8.26 (1.64)
Nurse	90	12.74 (1.49)	98	17.24 (1.96)	82	13.33 (1.67)	160	26.76 (2.18)	103	16.22 (1.78)	90	12.71 (1.49)
Public Health Science	170	11.78 (1.10)	219	15.05 (1.19)	216	14.23 (1.17)	343	25.32 (1.50)	218	14.30 (1.14)	173	11.52 (1.07)
*p*	*0.0534*		*0.0604*		*0.1137*		*0.1107*		*0.1608*		*0.2204*	
LHD												
Administrative	479	13.28 (0.86)	657	24.49 (1.32)	552	16.21 (1.03)	886	28.04 (1.05)	644	24.39 (1.22)	544	14.17 (0.87)
Clinical/BH/SS	452	13.04 (0.75)	607	16.36 (0.78)	546	15.01 (0.79)	861	27.07 (1.07)	634	17.69 (0.95)	438	11.49 (0.65)
Data/Informatics	64	8.08 (1.36)	65	7.62 (1.16)	45	4.30 (0.78)	125	21.74 (3.01)	74	8.23 (1.16)	53	6.57 (1.23)
Nurse	445	12.75 (0.76)	668	19.64 (0.92)	601	17.37 (0.87)	899	27.91 (1.08)	642	17.91 (0.87)	413	11.59 (0.70)
Public Health Science	650	12.48 (0.76)	840	17.66 (0.92)	768	13.89 (0.68)	1,230	28.48 (1.17)	921	18.92 (0.94)	627	11.77 (0.74)
*p*	*0.3485*		*0.0002*		*<0.0001*		*0.0332*		*<0.0001*		*0.061*	

SHA-CO, State Health Agency-Centralized Office; BCHD, Big City Health Department; LHD, other local health departments; BH, behavioral health; SS, social services; W%, Weighted Percent; SE, standard error.

Also presented in Table 1 are findings from the question on the self-reported skills gaps for the question, “Identify appropriate sources of data and information to assess the health of a community.” Workers in data/informatics-related roles reported the least training needs across all three settings: SHA-CO (8.5%), BCHC (9.3%) and LHD (7.6%). Administrative roles reported the highest skill gaps across all three settings: SHA-CO (24.7%), BCHC (20.1%) and LHD (24.5%).

The job category with the least self-reported skill gaps for the question “Collect valid data for use in decision making” was once again the data/informatics-related roles: SHA-CO (5.5%), BCHC (8.7%), and LHD (4.3%). The job categories with the highest reported skill gaps varied across settings with administrative roles (14.6%) in SHA-CO, PHS (14.2%) and Nurses in LHD (17.4%).

For the question on participation in quality improvement processes, the Public Health Science job category in LHDs expressed the most need (28.5%). Administrative job category indicated a higher need across all the 3 settings (25.4% in SHA-CO; 21.1% in BCHC; 24.4% in LHDs) for the question on identifying evidence-based approaches to address public health issues.

Table 1 data reveal significant skills gaps (*p* < 0.05) were observed for all six data/informatics topics in SHA-CO setting. Results suggest a significantly lower skill gap in one of the groups included in the analysis. In the BCHC setting, the only question with statistically significant result was pertaining to technical skills specific to program area (*p* = 0.0534). In the LHD setting, 4-out-of-6 data/informatics related questions were found to be statistically significant (*p* < 0.05), indicating that significant skills gaps were observed for these topics in one of the groups included in the analysis.

**Figure 3:**
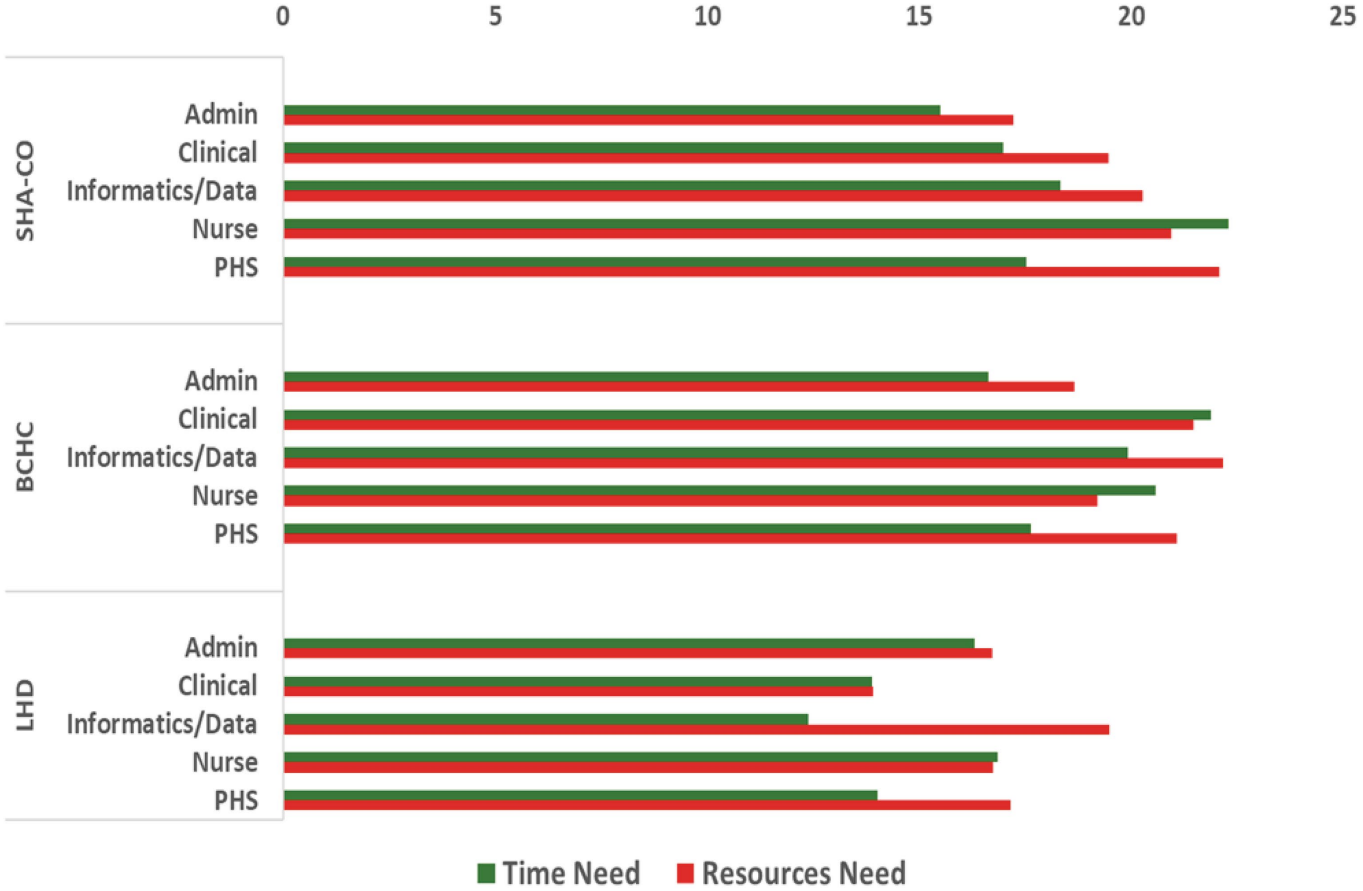
Time and resource needs for training by public health settings and job classifications among tier 1 governmental public health workers.

Figure 3 displays the perceived need for time and resources to address PH workers’ training needs based on the level of disagreement with availability. Nurses reported the highest need for time to meet training needs. Time as a factor was noted for nurses across all settings with more than one-fifth (22% in SHA-CO), followed by 21% in BCHC and 17% in LHD settings. The highest need for resources was reported by PHS job roles (22%) in SHA-CO, by data/informatics roles (22%) in BCHC, and by data/informatics roles (20%) in LHDs.

## 4 Discussion

This study offers insights into the skill gaps and training needs of the current Tier 1 (non-supervisory) US governmental workforce with a focus on data/informatics. The strength of the study is that it uses the most recent PH WINS survey data, the best available resource to enumerate the training needs of the governmental public health workforce. Broad and representative participation of public health agencies across the three settings (SHA-CO, BCHC and LHD), nationwide survey deployment in collaboration with key stakeholders, and rigorous survey methodology adds to the robustness of the study findings.

These results highlight a wide-spread need for training to address the informatics/technical skills gaps of the Tier 1 PH workforce and underscore the importance of continuous professional development in the digital era. The significant skill gaps observed for all data and information domains listed across the three settings (SHA-CO, BCHC, and LHD) is a finding which is similar to prior research (9). This underscores continued, unmet needs that should be addressed to support data modernization and digital transformation in public health. These findings are supported by similar results from the most recent NACCHO informatics profile which identified informatics as an area of staff training needs across PH roles (3). It is likely that these digital and technical skills gaps existed before the COVID-19 pandemic, but the pandemic exposed and exacerbated them (14). A notable finding is that close to half of the workforce has been <5 years in current position and majority without a public health degree, both of which hurdles to gaining contextual knowledge on the job.

Another noteworthy result is the need for time and resources for training across all three PH settings. Nurses emphasized the need for time to meet training needs. Prior PH WINS data (2022) suggest that public health and community health nurses were one of the largest occupation groups (15). The evident skill gap in this group warrants consideration, along with the noted need for time to meet training needs. Although many nursing schools provide informatics training in their curricula, it is unclear how many provide training in PH, data management, or PH informatics. A need on resources for training was expressed by all roles, with data/informatics roles expressing a high resource need across BCHC and LHD, which aligns with the NACCHO informatics profile report.

Prior research using 2022 PH WINS data had a limited inclusion criteria and compared the Public Health Informatics Specialist job role with other public health categories (9). This study also highlighted skill gaps and the need for training. The current study is more comprehensive in that it includes all the job classifications in the workforce and combines the various data-related positions into one category to create meaningful comparisons with the rest of the PH workforce.

One of the study limitations is that the results are based on analysis of self-reported data from respondents. Some positions which were managerial in nature were not included in this analysis as this research focused on Tier 1 respondents. Next steps of this research should be to focus on analysis of these roles as part of Tier 2 and 3 respondents. The varying level of skipped questions/missing responses needs to be accounted for in future studies. Another limitation is the absence of informatics-specific questions and so the identified digital skill gaps were based on questions which had data/technical orientation in fulfilling public health responsibilities. Questions on data / digital skills needs and informatics-driven public health functions (electronic data exchanges, systems requirements) should be added in future surveys.

The high indication of need for resources across all the job categories and across all the settings highlights the necessity of training to address the digital skill gaps. Informatics trainings with public health focus are being developed and expanded with some examples: PHI trainings (MPH, pre-doctoral and post-doctoral fellowships) at Indiana University (16), the CDC Public Health Informatics Fellowship Program (17), the CDC Data Science upskilling program (18), Data Science Team Training by CSTE (19) and the Public Health Informatics and Technology (PHIT) Workforce Development Program through the Assistant Secretary for Technology Policy (ASTP) (20). Expansion of these programs, along with partnerships between informatics programs and Schools of Public Health and Nursing should be encouraged to develop comprehensive, interdisciplinary curricula to address these skills gaps among current and future PH workers.

A workforce with data and technology-related skills are vital to support public health in this period of modernization and digital transformation. Multi-pronged training options along with adequate funding for workforce capacity building are essential to fill these skills gaps and upskill the current workforce to meet the evolving and emerging technical/digital skill requirements. Innovative approaches including strong academic—practice partnerships need to be envisioned to fill this void. Importantly, appropriate training needs to be designed and delivered to build an overall data and informatics-savvy workforce in public health practice.

## Data Availability

The data analyzed in this study is subject to the following licenses/restrictions: PH WINS data availability is restricted to select groups of researchers with study approvals through the de Beaumont Foundation. Requests to access these datasets should be directed to de Beaumont Foundation, phwins@debeaumont.org.
